# Poly(ionic liquid)-Modified Metal Organic Framework for Carbon Dioxide Adsorption

**DOI:** 10.3390/polym12020370

**Published:** 2020-02-07

**Authors:** Guangyuan Yang, Jialin Yu, Sanwen Peng, Kuang Sheng, Haining Zhang

**Affiliations:** 1 China Tobacco Hubei Industrial Cigarette Materials, LLC, Wuhan 430051, China; yangguangyuan@whut.edu.cn (G.Y.); shengk@163.com (K.S.); 2 State Key Laboratory of Advanced Technology for Materials Synthesis and Processing, Wuhan University of Technology, Wuhan 430070, China; zhn4you@yahoo.com

**Keywords:** metal organic framework, poly(ionic liquid), carbon dioxide, adsorption, imidazolium, temperature-programmed desorption

## Abstract

The design and synthesis of solid sorbents for effective carbon dioxide adsorption are essential for practical applications regarding carbon emissions. Herein, we report the synthesis of composite materials consisting of amine-functionalized imidazolium-type poly(ionic liquid) (PIL) and metal organic frameworks (MOFs) through complexation of amino groups and metal ions. The carbon dioxide adsorption behavior of the synthesized composite materials was evaluated using the temperature-programmed desorption (TPD) technique. Benefiting from the large surface area of metal organic frameworks and high carbon dioxide diffusivity in ionic liquid moieties, the carbon dioxide adsorption capacity of the synthesized composite material reached 19.5 cm^3^·g^−1^, which is much higher than that of pristine metal organic frameworks (3.1 cm^3^·g^−1^) under carbon dioxide partial pressure of 0.2 bar at 25 °C. The results demonstrate that the combination of functionalized poly(ionic liquid) with metal organic frameworks can be a promising solid sorbent for carbon dioxide adsorption.

## 1. Introduction

The unavoidable emission of CO_2_ generated by anthropogenic activities is one of the major contributors to the greenhouse effect and the accordingly induced environmental problems, particularly climate change and global warming [1,2,3]. Development of effective CO_2_ cleansing technologies is thus of great importance. Moreover, CO_2_ is also an important C1-chemical feedstock in different industrial fields, including chemical engineering, agriculture, and biosystems [4,5]. Capture through adsorption and further conversion of CO_2_ have become an important direction adopted in both academic and industrial areas.

Adsorption of CO_2_ by alkaline amine-based solutions is a commercially available technique that is often applied due to its excellent adsorption capacity [6,7]. However, the high volatility and decomposition of the applied alkaline amine and the serious corrosion to scrubbers have limited the practical applications [8,9,10]. Thus, the development of solid sorbents with a reasonable adsorption capacity and stability has become a widely investigated research area [11]. Particularly, amine-functionalized porous materials with a large surface area have been extensively investigated, and literature results have revealed that these materials are promising solid sorbents for CO_2_ adsorption [12,13,14,15,16,17,18,19,20]. Of the solid porous materials applied for CO_2_ adsorption, metal organic frameworks (MOFs) have attracted great attention due to their large surface area and the easy process for surface modification as pioneered by Yaghi [21,22,23,24,25,26,27,28] and well described in recently published topic reviews [29,30]. Among the developed MOF materials, Cu_3_(BTC)_2_ synthesized from Cu^2+^ and 1,3,5-benzenetricarboxylic acid (BTC) displays a promising ability for CO_2_ adsorption due to the large number of unsaturated active sites generated during thermal treatment for interaction with CO_2_ molecules [31]. The resulting CO_2_ storage capacity reached about 207 cm^3^·g^−1^ at 25.7 bar [31]. In addition, the exposed copper sites make the modification of the formed MOF possible through complexation with electron-rich atoms [32].

It has been reported that imidazolium-type ionic liquids exhibit high efficiency for CO_2_ capture due to the high solubility of CO_2_ caused by the formation of imidazolium-carbonate salts [33,34,35,36,37]. However, the dramatically increased viscosity of the reaction system after CO_2_ adsorption leads to the difficulty of mass transport of CO_2_ and the subsequent treatment for recycling [38]. This has led researchers’ interests to poly(ionic liquid) (PIL), which exhibits both the unique characteristics of ionic liquids and the physicochemical properties of polymers [39,40,41]. For example, Tang et al. reported that synthesized PIL showed an enhanced CO_2_ absorption capacity and fast adsorption/desorption rates compared to room-temperature ionic liquids [39].

Inspired by the advantages of MOFs and PIL on CO_2_ capture, amine-functionalized imidazolium-type PIL-modified Cu_3_(BTC)_2_ materials were designed and synthesized through complexation of amino groups on PIL with the exposed active sites of copper ions after thermal treatment. The idea behind this conceptual design was to improve the CO_2_ adsorption through the interaction of CO_2_ molecules with copper ions and ionic liquid moieties in addition to the physisorption in micropores of MOFs, and the application of the temperature-programmed desorption (TPD) technique was to differentiate the physisorption and chemisorption of CO_2_ molecules. The CO_2_ adsorption behavior of the thus synthesized composite material was evaluated under relatively mild conditions with the CO_2_ partial pressure of 0.2 bar at 25 °C by using the TPD technique. It is expected that the PIL-modified Cu_3_(BTC)_2_ can exhibit an improved CO_2_ adsorption capacity compared with Cu_3_(BTC)_2_ and pure PILs.

## 2. Materials and Methods

### 2.1. Materials

1-Vinylimidazole (1-VIm, purity of 99%), 2-bromoethanamine hydrobromide (98%), and 2,2-azobisisobutyronitrile (AIBN) were purchased from Alfa Aesar (MA, US). Prior to use, AIBN was recrystallized from methanol. Triethylamine (>99.5%), copper (II) nitrate hydrate (99%), and 1,3,5-benzenetricarboxylic acid (98%) were received from Aladdin (Shanghai, China). Deionized water with resistivity of 18.2 MΩ·cm^−1^ was generated from an Ulupure-H ultrapure water generator (Ulup, China). Unless otherwise specified, all the other solvents and reagents were used as received. The Cu_3_(BTC)_2_ was synthesized by following the literature [31].

### 2.2. Synthesis of Monomeric Ionic Liquid (VIm-NH_2_·HBr)

The monomeric ionic liquid was synthesized according to the literature with slight modification [42]. Briefly, in a two-neck flask, 1-VIm (9.41 g) was dissolved in absolute ethanol (50 mL). After refluxing under protection of argon atmosphere, 2-bromoethanamine hydrobromide (20.50 g) was added to the above solution. The mixture was continuously refluxed for 24 h and the resulting white precipitation was separated by centrifugation, followed by extensive washing with ethanol. The product (26.50 g) was obtained after drying under vacuum at 120 °C for 12 h. ^1^H NMR (D_2_O, 298 K, 300 MHz, ppm) δ: 7.92 (s, 1H, N=CH–N), 7.65 (s, 1H, N–CH=C), 7.13 (s, 1H, N–CH=C), 5.84 (dd, 2H, =CH2), 5.46 (dd, 1H, =CH), 4.77 (s, residue water in D_2_O), 4.58 (d, 1H, =N–CH–), 3.53 (t, 2H, C–CH_2_–N). FTIR (KBr pallet, cm^−1^): 3320, 3240, 3095, 3067, 2912, 1628, 1564, 1517, 1488, 1381, 1255, 1210, 1123.

### 2.3. Synthesis of Poly(ionic liquid) (PIL-NH_2_)

To a mixed solvent of *N*,*N*′-dimethylformamide (25 mL) and deionized water (10 mL), the synthesized VIm-NH2·HBr (3.98 g) and AIBN (4.0 mg) were added to form a homogenous solution. After removal of the dissolved oxygen in the solution by three freeze-thaw cycles, the solution was placed in a thermostat at 80 °C for polymerization and the polymerization time was set to 2 h. After polymerization, the resulting white precipitate was separated by centrifugation and was then dispersed in methanol (10 mL). Excessive triethylamine was added to the above dispersion to remove hydrobromide and the white solid was dissolved rapidly. The products were finally collected by precipitation after addition of ethyl acetate, followed by drying using a lyophilizer.

### 2.4. Synthesis of PIL-NH_2_-Modified Cu_3_(BTC)_2_ (Denoted as Cu_3_(BTC)_2_-PIL-NH_2_)

A 0.80 g amount of Cu_3_(BTC)_2_ was placed in a tube furnace and activated at 250 °C for 2.5 h, during which time the sample’s color turned from blue to purple. The activated sample of Cu_3_(BTC)_2_ was subsequently added to a solution of PIL-NHs (2.0 g) in methanol (5.0 mL) under protection of argon and the mixture was continuously stirred at room temperature for 24 h until the color of Cu_3_(BTC)_2_ became blue. The final composite product (0.91 g) was obtained after centrifugation and extensive washing with methanol, followed by drying at 70 °C under vacuum for 12 h.

### 2.5. Characterization

^1^H nuclear magnetic resonance (^1^H NMR, Mercury VX-300 spectrometer) was applied to determine the chemical structure of the synthesized monomer using tetramethylsilane as internal standard. Fourier-transform infrared (FTIR) spectra were recorded on a 60SXB spectrometer (Nicolet) in the range of 400–4000 cm^−1^ with a resolution of 4 cm^−1^ to qualitatively determine the synthesis of the corresponding products. Thermal stability of the samples was investigated by thermogravimetric analysis (TGA) and differential scan calorimetry (DSC) using an STA449F3 thermal analyzer (Netzsch) under a dynamic heating mode with ramp rate of 10 °C per minute in air over the temperature range of 30–1000 °C. X-ray diffraction (XRD) patterns were recorded from 5° to 70° to determine the crystal structure of Cu_3_(BTC)_2_ and Cu_3_(BTC)_2_-PIL-NH_2_ using Cu K_α_ irradiation sources with wavelength of 1.54 Å. The surface area and the porous structure of the samples were determined by nitrogen adsorption–desorption isotherms on a Micromeritics ASAP 2020 instrument at 77 K in the relative pressure range of 10^−8^ to 0.998. Prior to the measurements, samples were degassed at 120 °C for 6 h. The Brunauer–Emmett–Teller (BET) model was applied to calculate specific surface area and the Barrett–Joyner–Halenda (BJH) approach was taken to obtain the pore size distribution. X-ray photoelectron spectroscopy (XPS, VG Multilab2000X, Al K_α_ irradiation source) was applied to determine the elemental composition near the surface of samples.

### 2.6. CO_2_ Adsorption

The CO_2_ adsorption behavior was performed on a TPD apparatus TP-5080 (Tianjin Xianquan, China) using helium as carrier gas. The typical procedure includes pre-adsorption of CO_2_ at desired temperature under CO_2_ partial pressure of 0.2 bar, followed by temperature-dependent desorption and finally complete desorption of the adsorbed CO_2_ molecules at 200 °C. The detailed procedure was described elsewhere [43]. The adsorbed amount of CO_2_ was calculated from the detected TPD signals of thermal conductivity detector using the software from the supplier.

## 3. Results

**Figure 1:**
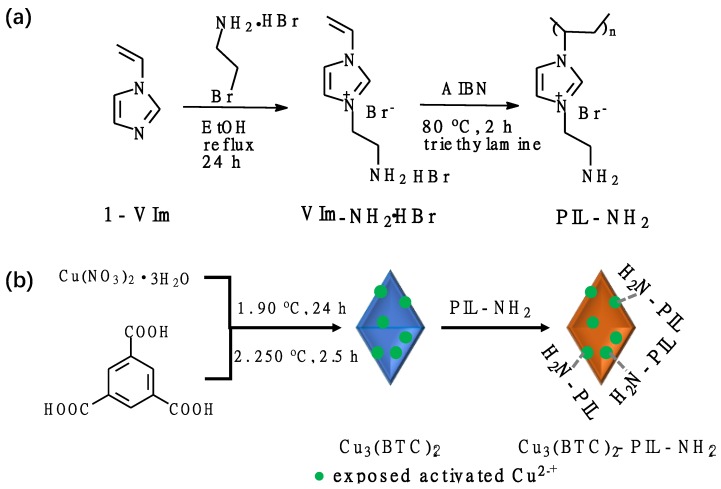
(**a**) Synthesis of amine-functionalized imidazolium-type poly(ionic liquid). (**b**) Schematic illustration of the synthetic process of composite sorbent of Cu_3_(BTC)_2_ and poly(ionic liquid).

The idea behind the design of the composite materials for CO_2_ adsorption was to take the advantages of the large surface area of MOF materials and the great CO_2_ adsorption capacity of amine-functionalized imidazolium-type poly(ionic liquid)s. Thus, the designed amine-functionalized poly(ionic liquid) (PIL-NH_2_) was first synthesized through simple free-radical polymerization, as illustrated in Figure 1a. The amino groups on the synthesized PIL-NH_2_ can therefore interact with the exposed copper ions on the preformed metal organic framework through complexation, leading to the modification of Cu_3_(BTC)_2_ by poly(ionic liquid), as shown in Figure 1b.

**Figure 2:**
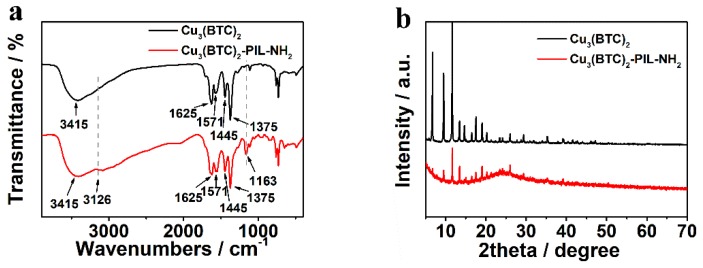
(**a**) FTIR spectra and (**b**) XRD patterns of Cu_3_(BTC)_2_ and Cu_3_(BTC)_2_-PIL-NH_2_ as indicated in the figure. Dashed lines in the FTIR spectrum are a visual guide.

The FTIR spectrum was first recorded to monitor the interaction of Cu_3_(BTC)_2_ and poly(ionic liquid), as shown in Figure 2a. For better comparison, the FTIR spectrum of Cu_3_(BTC)_2_ was plotted in the same figure. In both FTIR spectra, the characteristic absorption bands for benzene rings from Cu_3_(BTC)_2_ can be clearly observed at 1625 and 1571 cm^−1^ [31]. The absorption bands at 1375 and 1280 cm^−1^ are assigned to the bending vibration of hydroxy groups of COOH and the stretching vibration of C–O, respectively. Compared to the spectrum of Cu_3_(BTC)_2_, two new absorption bands appeared at 3126 and 1163 cm^−1^ in the FTIR spectrum of Cu_3_(BTC)_2_-PIL-NH_2_ assigned to the stretching vibration of =C–H and the stretching vibration of C–N of the imidazole rings, indicating the successful complex formation [41]. Figure 2b displays the XRD patterns of Cu_3_(BTC)_2_ and Cu_3_(BTC)_2_-PIL-NH_2_. It can be seen that the diffraction peaks are very sharp for Cu_3_(BTC)_2_, suggesting the great crystallinity. In addition, the position of diffraction peaks agrees well with those reported in the literature [31], indicating that crystallized Cu_3_(BTC)_2_ was successfully synthesized. For Cu_3_(BTC)_2_-PIL-NH_2_, diffraction peaks were observed at the same angles of Cu_3_(BTC)_2_ with decreased intensity and a new broad diffraction peak appeared at 2θ of 15–30°, revealing the existence of amorphous poly(ionic liquid)s and the partially destroyed crystalline structure of Cu_3_(BTC)_2_.

**Figure 3:**
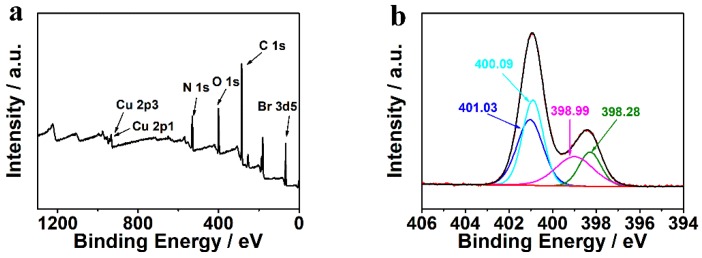
(**a**) Full XPS survey and (**b**) high-resolution N 1s XPS spectrum of Cu_3_(BTC)_2_-PIL-NH_2_. The deconvolution results of N 1s peak are also displayed in (**b**).

Figure 3 shows XPS results of the synthesized Cu_3_(BTC)_2_-PIL-NH_2_ composite material. The full XPS survey (Figure 3a) clearly suggests the existence of atoms including Cu (781.2 eV for Cu 2p), N (400.8 eV for N 1s), O (531.3 eV for O 1s), C (285.6 eV for C 1s), and Br (67.7 eV for Br 3d), further confirming the formation of composite materials. To evaluate the bonding formation between Cu_3_(BTC)_2_ and PIL-NH_2_, the high-resolution N 1s peak was deconvoluted, as shown in Figure 3b. It can be seen that N atoms in the composite material have four different bonding states with the binding energy centered at 398.28, 398.99, 400.09, and 401.03 eV. It has been reported that the peaks centered at 400.09 and 401.03 eV corresponded to the C–N and C=N bonding on the imidazole rings of ionic liquid moieties [28]. The peaks at 398.28 and 398.99 eV can be therefore assigned to amino groups. It is expected that two types of amino groups exist in the designed composite materials as free amino groups and the bonded amino groups with copper ions. Since the complexation of amino groups with transition metal ions can lead to reduced binding energy, the peak centered at 398.28 should correspond to amino groups interacted with copper ions and the peak centered at 398.99 is related to free amino groups in the composite material.

**Figure 4:**
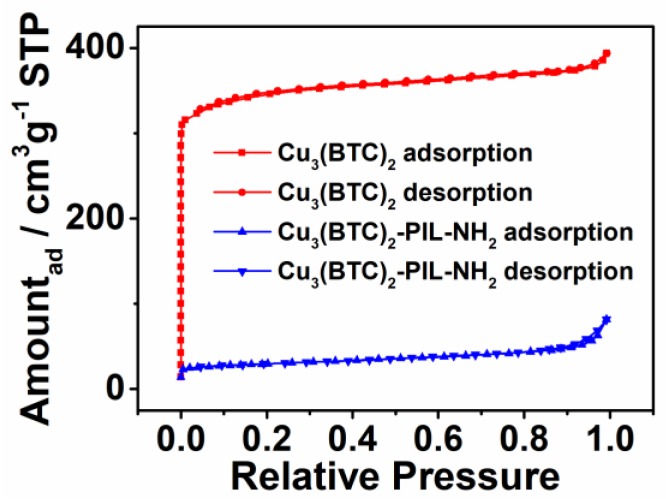
Nitrogen adsorption–desorption isotherms for Cu_3_(BTC)_2_ and Cu_3_(BTC)_2_-PIL-NH_2_.

The porous structure of sorbents has significant influence on their adsorption behavior of gaseous species. Nitrogen adsorption/desorption isotherms were therefore recorded to determine the porous structure of Cu_3_(BTC)_2_-PIL-NH_2_ and Cu_3_(BTC)_2_, as shown in Figure 4. It is apparent that Cu_3_(BTC)_2_ exhibited the typical Type I isotherms, suggesting the existence of micropores. With the grafting of PILs onto the sample’s surface, the shape of the isotherm changed significantly, particularly under the low relative pressure, suggesting the significant decrease in numbers of micropores due to the blockage of surface-attached polymer chains. The derived porous parameters including surface area, pore volume, and average pore size are listed in Table 1. It can be seen that Cu_3_(BTC)_2_ has a surface area of 1352 cm^2^·g^−1^ with average pore size of 1.8 nm and pore volume of 0.61 cm^3^·g^−1^. However, both surface area and pore volume dramatically decreased to 107 m^2^·g^−1^ and 0.12 cm^3^·g^−1^, respectively. This could be attributed to the tensive coverage of micropores on Cu_3_(BTC)_2_ by the introduced polymer chains. The increased average pore diameter of the sample after complexation with PIL-NH_2_ is possibly related to slit pores or the formed pores due to accumulation of the PIL-NH_2_-modified particles.

**Figure 5:**
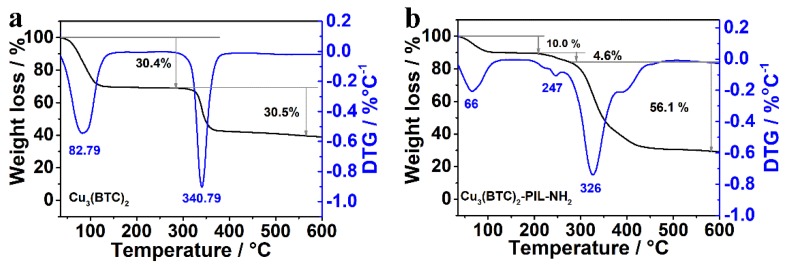
(**a**) TG (black) and DTG (blue) curves of Cu_3_(BTC)_2_. (**b**) TG (black) and DTG (blue) curves of Cu_3_(BTC)_2_-PIL-NH_2_. The corresponding weight loss values are listed in the figure.

Since CO_2_ adsorption was investigated using the temperature-programmed desorption (TPD) technique, the thermal stability of sorbents is one essential parameter to be achieved. Figure 5 shows the TG and DTG curves of Cu_3_(BTC)_2_ and Cu_3_(BTC)_2_-PIL-NH_2_. It can be seen that both samples have apparent weight losses under temperatures below 120 °C and under temperatures above 275 °C, corresponding to the release of the physically adsorbed water molecules and the decomposition of organic parts (BTC and PIL), respectively. Compared with Cu_3_(BTC)_2_, Cu_3_(BTC)_2_-PIL-NH_2_ exhibits one additional weight loss region between 210 and 275 °C, possibly attributed to the release of structured water molecules that are interacted with N atoms in PIL through hydrogen bonding. It can be thus concluded that the synthesized Cu_3_(BTC)_2_-PIL-NH_2_ is thermally stable at temperatures below 200 °C and the operational temperature for TPD measurements can be set to 200 °C.

**Figure 6:**
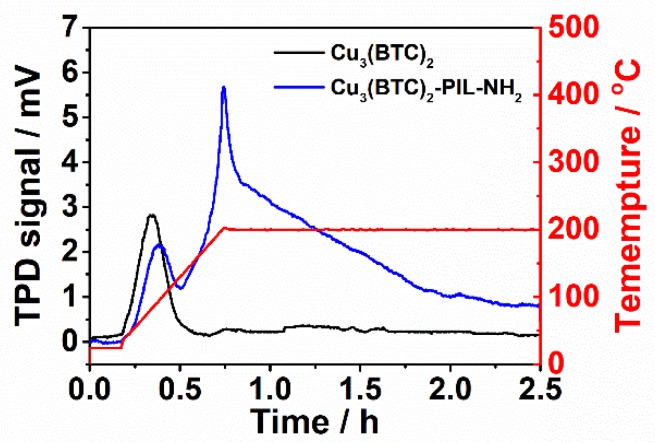
CO_2_ TPD response curves for Cu_3_(BTC)_2_ and Cu_3_(BTC)_2_-PIL-NH_2_. Red line in the figure refers to the temperature profile for desorption.

The CO_2_ adsorption behavior was determined by desorption (TPD technique) under the temperature range of 25 to 200 °C of pre-adsorbed CO_2_ molecules in the samples at 25 °C under CO_2_ pressure of 0.2 bar for 2 h. The TPD response curves for Cu_3_(BTC)_2_ and Cu_3_(BTC)_2_-PIL-NH_2_ are plotted in Figure 6. It is evident that only one desorption peak at about 95 °C was observed for Cu_3_(BTC)_2_. The corresponding calculated CO_2_ adsorption capacity is about 3.09 cm^3^·g^−1^. The observed desorption peak can be therefore assigned to the desorption of physically adsorbed CO_2_ molecules and the adsorbed CO_2_ molecules through weak interaction with copper ions in Cu_3_(BTC)_2_ [31]. For Cu_3_(BTC)_2_-PIL-NH_2_, there are two desorption peaks at about 96 and 200 °C, respectively. Similar to that for Cu_3_(BTC)_2_, the desorption peak at 96 °C can be attributed to the release of physically adsorbed CO_2_ molecules. The physically adsorbed amount is about 2.24 cm^3^·g^−1^, smaller than that of Cu_3_(BTC)_2_ due to the decreased surface area. It is thus hypothesized that the desorption at temperatures above 100 °C could be attributed to the chemical adsorption of CO_2_ molecules induced by the presence of PIL-NH_2_ chains as the release of chemically adsorbed CO_2_ molecules requires more energy. Since both amino groups and 2-position carbon atoms in the imidazole ring can be chemically interacted with CO_2_ molecules [36,37,41], the Cu_3_(BTC)_2_-PIL-NH_2_ exhibited a considerably large chemical adsorption capacity of about 17.3 cm^3^·g^−1^.

## 4. Discussion

**Figure 7:**
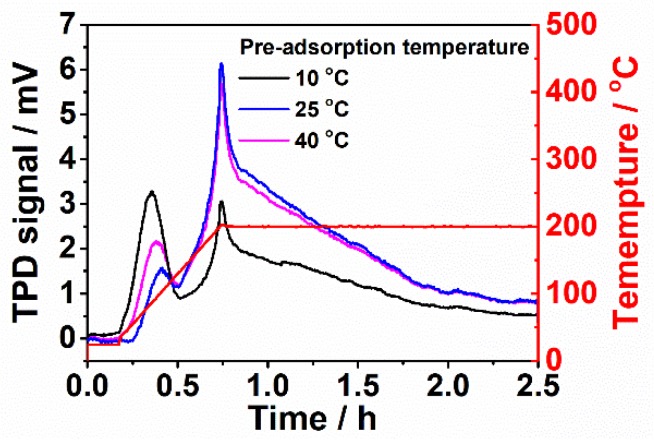
CO_2_ TPD response curves of Cu_3_(BTC)_2_-PIL-NH_2_ for pre-adsorption of CO_2_ at different temperatures. Red line in the figure refers to the temperature profile for desorption.

To validate the hypothesis on the physisorption and chemisorption of CO_2_ using Cu_3_(BTC)_2_-PIL-NH_2_ as sorbent, the adsorption of CO_2_ at different temperatures was performed and the same TPD process was applied. The CO_2_ TPD response curves are displayed in Figure 7. In general, the physisorption capacity of gaseous species decreased with the increase in temperature due to the existing equilibrium of adsorption and desorption, whereas the chemisorption capacity increased with the increase in temperature because of the enhanced reaction kinetics [11,13,21]. It is evident that the peak intensity at temperature of about 95 °C decreased and the peak intensity at about 200 °C increased while the adsorption temperature increased from 10 to 40 °C. The corresponding desorbed amount at 95 °C calculated from TPD curves decreased from 3.4 to 1.4 cm^3^·g^−1^ with the increase in adsorption temperature from 10 to 40 °C, suggesting that the desorption at 95 °C resulted from the physically adsorbed CO_2_. In contrast, the desorbed amount at 200 °C increased from 14.6 to 17.6 cm^3^·g^−1^ with the increase in adsorption temperature from 10 to 40 °C, indicating that the desorption at 200 °C resulted from the chemisorption of CO_2_ molecules through the interaction between CO_2_ molecules with amino groups and 2-position carbon atoms of imidazolium-type poly(ionic liquid)s. It should be mentioned that the chemically adsorbed amount at 40 °C is only slightly higher than that at 25 °C. The total CO_2_ adsorption capacity at 25 °C (19.5 cm^3^·g^−1^) is higher than that at 40 °C (19.0 cm^3^·g^−1^) due to the good physical adsorption. It should also be mentioned that the total CO_2_ adsorption capacity of Cu_3_(BTC)_2_-PIL-NH_2_ derived from the TPD technique is still quite low for practical applications due to the possibly slow adsorption kinetics and the limitation of the apparatus which cannot be applied at high pressure. It is expected that the CO_2_ adsorption capacity can be greatly improved with the increase in operational pressures with which physical adsorption of CO_2_ on porous materials can be significantly enhanced.

## 5. Conclusions

In summary, anamine-functionalized imidazolium-type poly(ionic liquid)-modified metal organic framework was synthesized as sorbent for carbon dioxide adsorption via complexation of amino groups on polymer chains and metal ions. After modification with functionalized poly(ionic liquid) chains, the surface area of the metal organic framework reduced significantly, leading to a decreased physical adsorption capacity of carbon dioxide molecules. However, the chemical adsorption through interaction of amino groups and 2-position carbon atoms on the imidazolium rings with carbon dioxide dominates the total adsorption capacity under the relatively low carbon dioxide pressure. Moreover, the physisorption and chemisorption of carbon dioxide on the synthesized composite sorbents can be easily identified using the temperature-programmed desorption technique. The results in this work demonstrate that the modification of porous materials with amine-functionalized imidazolium-type poly(ionic liquid)s can be a promising approach in the design and synthesis of model sorbents for efficient carbon dioxide adsorption by improving the physical adsorption kinetics under high pressure.

## Figures and Tables

**Table 1:** The derived porous parameters of Cu_3_(BTC)_2_ and Cu_3_(BTC)_2_-PIL-NH_2_.

Samples	Surface Area (m^2^·g^−1^)	Pore Volume (cm^3^·g^−1^)	Pore Size (nm)
Cu_3_(BTC)_2_	1352	0.60	1.8
Cu_3_(BTC)_2_-PIL-NH_2_	107	0.12	4.5
